# Supplementary material for: HIVprotI: an integrated web based platform for prediction and design of HIV proteins inhibitors
Source: J Cheminform. 2018 Mar 9;10:12. doi: 10.1186/s13321-018-0266-y (PMC5845081; doi:10.1186/s13321-018-0266-y)
Supplement: Supplementary file 2 — Additional file 2. Source code of HIVProtI web server. [file 13321_2018_266_MOESM2_ESM.zip › HIVprotI_Source-code/hivprotI/weka-3-6-6/doc/weka/gui/beans/ClassifierPerformanceEvaluator.html]

ClassifierPerformanceEvaluator


---


|  |  |  |
| --- | --- | --- |
| **Overview** \| **Package** \| **Class** \| **Tree** \| **Deprecated** \| **Index** \| **Help** \| **Weka's home** | |  |
| **PREV CLASS**   **NEXT CLASS** | **FRAMES**    **NO FRAMES**     **All Classes** |  |
| SUMMARY: NESTED \| FIELD \| CONSTR \| METHOD | DETAIL: FIELD \| CONSTR \| METHOD |  |


---


## weka.gui.beans Class ClassifierPerformanceEvaluator

```
java.lang.Object
  java.awt.Component
      java.awt.Container
          javax.swing.JComponent
              javax.swing.JPanel
                  weka.gui.beans.AbstractEvaluator
                      weka.gui.beans.ClassifierPerformanceEvaluator
```

**All Implemented Interfaces:**: java.awt.image.ImageObserver, java.awt.MenuContainer, java.io.Serializable, java.util.EventListener, javax.accessibility.Accessible, BatchClassifierListener, BeanCommon, EventConstraints, UserRequestAcceptor, Visible

---

``` public class ClassifierPerformanceEvaluator extends AbstractEvaluator implements BatchClassifierListener, java.io.Serializable, UserRequestAcceptor, EventConstraints ```

A bean that evaluates the performance of batch trained classifiers

**Version:**
:   $Revision: 7059 $

**Author:**
:   Mark Hall

**See Also:**: Serialized Form

---

**Nested Class Summary**

**Nested classes/interfaces inherited from class javax.swing.JComponent**

`javax.swing.JComponent.AccessibleJComponent`

**Nested classes/interfaces inherited from class java.awt.Component**

`java.awt.Component.BaselineResizeBehavior`


**Field Summary**

**Fields inherited from class javax.swing.JComponent**

`TOOL_TIP_TEXT_KEY, UNDEFINED_CONDITION, WHEN_ANCESTOR_OF_FOCUSED_COMPONENT, WHEN_FOCUSED, WHEN_IN_FOCUSED_WINDOW`

**Fields inherited from class java.awt.Component**

`BOTTOM_ALIGNMENT, CENTER_ALIGNMENT, LEFT_ALIGNMENT, RIGHT_ALIGNMENT, TOP_ALIGNMENT`

**Fields inherited from interface java.awt.image.ImageObserver**

`ABORT, ALLBITS, ERROR, FRAMEBITS, HEIGHT, PROPERTIES, SOMEBITS, WIDTH`


**Constructor Summary**

`ClassifierPerformanceEvaluator()`


| **Method Summary** | |
| --- | --- |
| `void` | `acceptClassifier(BatchClassifierEvent ce)`             Accept a classifier to be evaluated |
| `void` | `addTextListener(TextListener cl)`             Add a text listener |
| `void` | `addThresholdDataListener(ThresholdDataListener cl)`             Add a threshold data listener |
| `void` | `addVisualizableErrorListener(VisualizableErrorListener vel)`             Add a visualizable error listener |
| `java.util.Enumeration` | `enumerateRequests()`             Return an enumeration of user activated requests for this bean |
| `boolean` | `eventGeneratable(java.lang.String eventName)`             Returns true, if at the current time, the named event could be generated. |
| `java.lang.String` | `getCustomName()`             Get the custom (descriptive) name for this bean (if one has been set) |
| `java.lang.String` | `globalInfo()`             Global info for this bean |
| `boolean` | `isBusy()`             Returns true if. |
| `void` | `performRequest(java.lang.String request)`             Perform the named request |
| `void` | `removeTextListener(TextListener cl)`             Remove a text listener |
| `void` | `removeThresholdDataListener(ThresholdDataListener cl)`             Remove a Threshold data listener |
| `void` | `removeVisualizableErrorListener(VisualizableErrorListener vel)`             Remove a visualizable error listener |
| `void` | `setCustomName(java.lang.String name)`             Set a custom (descriptive) name for this bean |
| `void` | `stop()`             Try and stop any action |

**Methods inherited from class weka.gui.beans.AbstractEvaluator**

`connectionAllowed, connectionAllowed, connectionNotification, disconnectionNotification, getVisual, setLog, setVisual, useDefaultVisual`

**Methods inherited from class javax.swing.JPanel**

`getAccessibleContext, getUI, getUIClassID, setUI, updateUI`

**Methods inherited from class javax.swing.JComponent**

`addAncestorListener, addNotify, addVetoableChangeListener, computeVisibleRect, contains, createToolTip, disable, enable, firePropertyChange, firePropertyChange, firePropertyChange, getActionForKeyStroke, getActionMap, getAlignmentX, getAlignmentY, getAncestorListeners, getAutoscrolls, getBaseline, getBaselineResizeBehavior, getBorder, getBounds, getClientProperty, getComponentPopupMenu, getConditionForKeyStroke, getDebugGraphicsOptions, getDefaultLocale, getFontMetrics, getGraphics, getHeight, getInheritsPopupMenu, getInputMap, getInputMap, getInputVerifier, getInsets, getInsets, getListeners, getLocation, getMaximumSize, getMinimumSize, getNextFocusableComponent, getPopupLocation, getPreferredSize, getRegisteredKeyStrokes, getRootPane, getSize, getToolTipLocation, getToolTipText, getToolTipText, getTopLevelAncestor, getTransferHandler, getVerifyInputWhenFocusTarget, getVetoableChangeListeners, getVisibleRect, getWidth, getX, getY, grabFocus, isDoubleBuffered, isLightweightComponent, isManagingFocus, isOpaque, isOptimizedDrawingEnabled, isPaintingForPrint, isPaintingTile, isRequestFocusEnabled, isValidateRoot, paint, paintImmediately, paintImmediately, print, printAll, putClientProperty, registerKeyboardAction, registerKeyboardAction, removeAncestorListener, removeNotify, removeVetoableChangeListener, repaint, repaint, requestDefaultFocus, requestFocus, requestFocus, requestFocusInWindow, resetKeyboardActions, reshape, revalidate, scrollRectToVisible, setActionMap, setAlignmentX, setAlignmentY, setAutoscrolls, setBackground, setBorder, setComponentPopupMenu, setDebugGraphicsOptions, setDefaultLocale, setDoubleBuffered, setEnabled, setFocusTraversalKeys, setFont, setForeground, setInheritsPopupMenu, setInputMap, setInputVerifier, setMaximumSize, setMinimumSize, setNextFocusableComponent, setOpaque, setPreferredSize, setRequestFocusEnabled, setToolTipText, setTransferHandler, setVerifyInputWhenFocusTarget, setVisible, unregisterKeyboardAction, update`

**Methods inherited from class java.awt.Container**

`add, add, add, add, add, addContainerListener, addPropertyChangeListener, addPropertyChangeListener, applyComponentOrientation, areFocusTraversalKeysSet, countComponents, deliverEvent, doLayout, findComponentAt, findComponentAt, getComponent, getComponentAt, getComponentAt, getComponentCount, getComponents, getComponentZOrder, getContainerListeners, getFocusTraversalKeys, getFocusTraversalPolicy, getLayout, getMousePosition, insets, invalidate, isAncestorOf, isFocusCycleRoot, isFocusCycleRoot, isFocusTraversalPolicyProvider, isFocusTraversalPolicySet, layout, list, list, locate, minimumSize, paintComponents, preferredSize, printComponents, remove, remove, removeAll, removeContainerListener, setComponentZOrder, setFocusCycleRoot, setFocusTraversalPolicy, setFocusTraversalPolicyProvider, setLayout, transferFocusBackward, transferFocusDownCycle, validate`

**Methods inherited from class java.awt.Component**

`action, add, addComponentListener, addFocusListener, addHierarchyBoundsListener, addHierarchyListener, addInputMethodListener, addKeyListener, addMouseListener, addMouseMotionListener, addMouseWheelListener, bounds, checkImage, checkImage, contains, createImage, createImage, createVolatileImage, createVolatileImage, dispatchEvent, enable, enableInputMethods, firePropertyChange, firePropertyChange, firePropertyChange, firePropertyChange, firePropertyChange, getBackground, getBounds, getColorModel, getComponentListeners, getComponentOrientation, getCursor, getDropTarget, getFocusCycleRootAncestor, getFocusListeners, getFocusTraversalKeysEnabled, getFont, getForeground, getGraphicsConfiguration, getHierarchyBoundsListeners, getHierarchyListeners, getIgnoreRepaint, getInputContext, getInputMethodListeners, getInputMethodRequests, getKeyListeners, getLocale, getLocation, getLocationOnScreen, getMouseListeners, getMouseMotionListeners, getMousePosition, getMouseWheelListeners, getName, getParent, getPeer, getPropertyChangeListeners, getPropertyChangeListeners, getSize, getToolkit, getTreeLock, gotFocus, handleEvent, hasFocus, hide, imageUpdate, inside, isBackgroundSet, isCursorSet, isDisplayable, isEnabled, isFocusable, isFocusOwner, isFocusTraversable, isFontSet, isForegroundSet, isLightweight, isMaximumSizeSet, isMinimumSizeSet, isPreferredSizeSet, isShowing, isValid, isVisible, keyDown, keyUp, list, list, list, location, lostFocus, mouseDown, mouseDrag, mouseEnter, mouseExit, mouseMove, mouseUp, move, nextFocus, paintAll, postEvent, prepareImage, prepareImage, remove, removeComponentListener, removeFocusListener, removeHierarchyBoundsListener, removeHierarchyListener, removeInputMethodListener, removeKeyListener, removeMouseListener, removeMouseMotionListener, removeMouseWheelListener, removePropertyChangeListener, removePropertyChangeListener, repaint, repaint, repaint, resize, resize, setBounds, setBounds, setComponentOrientation, setCursor, setDropTarget, setFocusable, setFocusTraversalKeysEnabled, setIgnoreRepaint, setLocale, setLocation, setLocation, setName, setSize, setSize, show, show, size, toString, transferFocus, transferFocusUpCycle`

**Methods inherited from class java.lang.Object**

`equals, getClass, hashCode, notify, notifyAll, wait, wait, wait`

**Constructor Detail**

### ClassifierPerformanceEvaluator

```
public ClassifierPerformanceEvaluator()
```


**Method Detail**

### setCustomName

```
public void setCustomName(java.lang.String name)
```

:   Set a custom (descriptive) name for this bean

    :   **Specified by:**: `setCustomName` in interface `BeanCommon`
    :   **Parameters:**: `name` - the name to use

---


### getCustomName

```
public java.lang.String getCustomName()
```

:   Get the custom (descriptive) name for this bean (if one has been set)

    :   **Specified by:**: `getCustomName` in interface `BeanCommon`
    :   **Returns:**: the custom name (or the default name)

---


### globalInfo

```
public java.lang.String globalInfo()
```

:   Global info for this bean

    :   **Returns:**: a `String` value

---


### acceptClassifier

```
public void acceptClassifier(BatchClassifierEvent ce)
```

:   Accept a classifier to be evaluated

    :   **Specified by:**: `acceptClassifier` in interface `BatchClassifierListener`
    :   **Parameters:**: `ce` - a `BatchClassifierEvent` value

---


### isBusy

```
public boolean isBusy()
```

:   Returns true if. at this time, the bean is busy with some
    (i.e. perhaps a worker thread is performing some calculation).

    :   **Specified by:**: `isBusy` in interface `BeanCommon`
    :   **Returns:**: true if the bean is busy.

---


### stop

```
public void stop()
```

:   Try and stop any action

    :   **Specified by:**: `stop` in interface `BeanCommon` **Specified by:**: `stop` in class `AbstractEvaluator`

---


### enumerateRequests

```
public java.util.Enumeration enumerateRequests()
```

:   Return an enumeration of user activated requests for this bean

    :   **Specified by:**: `enumerateRequests` in interface `UserRequestAcceptor`
    :   **Returns:**: an `Enumeration` value

---


### performRequest

```
public void performRequest(java.lang.String request)
```

:   Perform the named request

    :   **Specified by:**: `performRequest` in interface `UserRequestAcceptor`
    :   **Parameters:**: `request` - the request to perform **Throws:**: `java.lang.IllegalArgumentException` - if an error occurs

---


### addTextListener

```
public void addTextListener(TextListener cl)
```

:   Add a text listener

    :   **Parameters:**: `cl` - a `TextListener` value

---


### removeTextListener

```
public void removeTextListener(TextListener cl)
```

:   Remove a text listener

    :   **Parameters:**: `cl` - a `TextListener` value

---


### addThresholdDataListener

```
public void addThresholdDataListener(ThresholdDataListener cl)
```

:   Add a threshold data listener

    :   **Parameters:**: `cl` - a `ThresholdDataListener` value

---


### removeThresholdDataListener

```
public void removeThresholdDataListener(ThresholdDataListener cl)
```

:   Remove a Threshold data listener

    :   **Parameters:**: `cl` - a `ThresholdDataListener` value

---


### addVisualizableErrorListener

```
public void addVisualizableErrorListener(VisualizableErrorListener vel)
```

:   Add a visualizable error listener

    :   **Parameters:**: `vel` - a `VisualizableErrorListener` value

---


### removeVisualizableErrorListener

```
public void removeVisualizableErrorListener(VisualizableErrorListener vel)
```

:   Remove a visualizable error listener

    :   **Parameters:**: `vel` - a `VisualizableErrorListener` value

---


### eventGeneratable

```
public boolean eventGeneratable(java.lang.String eventName)
```

:   Returns true, if at the current time, the named event could
    be generated. Assumes that supplied event names are names of
    events that could be generated by this bean.

    :   **Specified by:**: `eventGeneratable` in interface `EventConstraints`
    :   **Parameters:**: `eventName` - the name of the event in question **Returns:**: true if the named event could be generated at this point in time


---


|  |  |  |
| --- | --- | --- |
| **Overview** \| **Package** \| **Class** \| **Tree** \| **Deprecated** \| **Index** \| **Help** \| **Weka's home** | |  |
| **PREV CLASS**   **NEXT CLASS** | **FRAMES**    **NO FRAMES**     **All Classes** |  |
| SUMMARY: NESTED \| FIELD \| CONSTR \| METHOD | DETAIL: FIELD \| CONSTR \| METHOD |  |


---
